# Quality of life of women with breast cancer: A cross sectional study in a regional hospital

**DOI:** 10.1192/j.eurpsy.2023.2000

**Published:** 2023-07-19

**Authors:** F. Zaouali, I. Abbes, A. Ben Slama, I. Belhaj Youssef

**Affiliations:** 1 Outpatient Medical Oncology consultation, Haj Ali Soua regional hospital, Ksar Hellal, Monastir; 2 Family Medicine Department, University of Monastir, Monastir; 3 Outpatient Physical Medicine and Rehabilitation consultation, Haj Ali Soua regional hospital, Ksar Hellal, Monastir, Tunisia

## Abstract

**Introduction:**

The assessment of quality of life is an essential complement to medical care. Some studies have shown that young women are more vulnerable to the disease impact and have a greater worsening of their quality of life.

**Objectives:**

The aim of our study was to assess the quality of life of patients with breast cancer.

**Methods:**

Cross-sectional descriptive study including patients followed for breast cancer at the outpatient medical oncology consultation of Hadj Ali Soua regional hospital from January to March 2021. We applied the 36-Item Short Form Survey SF-36.

**Results:**

Fifteen patients were included with a mean age of 49.87 ± 8.48 years and a mean age at diagnosis of 46.73 ± 7.55 years. At the TNM classification, 66.6% of the patients had a T1 or T2 at the time of diagnosis and 80% had an N0. All patients received a surgical intervention, which was conservative in 53.3% of cases. No patient underwent breast reconstruction. Chemotherapy and hormone therapy were prescribed in 86.7% of patients. There was unequal impairment of different areas of the SF-36 questionnaire. The physical component was the most affected with a mean physical score (PCS) of 62.64; the RP score (limitations due to physical condition) was the lowest with a mean of 45 and the score of the item “Life and relations with others” was the best with a mean of 77.5.

**Conclusions:**

The quality of life in relation to breast cancer in our population was at the medium rating, with moderately high scores. Sustainable improvement of the quality of life of women with breast cancer is a priority issue among the treatment objectives. Further studies are needed to assess the impact on the spouse, which is inseparable from the couple.

**Disclosure of Interest:**

None Declared

